# Outcome associations of CSF total tau in suspected non-Alzheimer pathophysiology

**DOI:** 10.1007/s00415-026-13841-x

**Published:** 2026-04-30

**Authors:** Nils Briel, Marc Borsi, Simon J. Schreiner, Thomas Schneider, Sandra V. Loosli, Claudio Togni, Manfredi C. Carta, Jan Loosli, Alexander Köpp, Mario Ziegler, Tommaso Nicoletti, Michael Weller, Ansgar Felbecker, Tobias Weiss, Hans H. Jung

**Affiliations:** 1 Department of Neurology, University Hospital Zurich and University of Zurich, Frauenklinikstrasse 26, CH-8091 Zurich, Switzerland; 2 Department of Chemistry and Applied Biosciences (D-CHAB), ETH Zurich, Vladimir-Prelog-Weg 1-5/10, CH-8093 Zurich, Switzerland; 3 Department of Neurology, HOCH Health Ostschweiz, Rorschacher Strasse 95, CH-9007 St. Gallen, Switzerland; 4 Division of Clinical Geriatrics, Department of Neurobiology, Care Sciences and Society, Center for Alzheimer Research, Karolinska Institutet, 141 52 Huddinge, Sweden; 5 Department of Neuropathology, Pitié-Salpêtrière Hospital, AP-HP Sorbonne University, Paris, France; 6 Institut du Cerveau, Paris Brain Institute, ICM, Inserm U1127, CNRS UMR7225, APHP, Sorbonne University, Pitié-Salpêtrière Hospital, Paris, France; 7 Department of Neurology, Inselspital, University Hospital Bern, Rosenbühlgasse 25, CH-3010 Bern, Switzerland; 8 Neuroscience Center Zurich University of Zurich, Zurich, Switzerland 8057

**Keywords:** SNAP, Alzheimer’s disease, Tau, Mortality, Survival, Prognostic biomarker

## Abstract

**Introduction:**

Cognitively impaired patients with negative biomarkers of amyloidosis but with neurochemical evidence of Tau pathophysiology or neurodegeneration or both are classified as “suspected non-Alzheimer pathophysiology” (SNAP), according to the 2018 research framework for a biological definition of Alzheimer’s disease (AD). The SNAP concept remains incompletely characterized, and associations of amyloid and tau biomarkers with survival outcomes in the SNAP context remain unclear.

**Methods:**

We conducted a retrospective study in patients with a SNAP biomarker constellation, recruited from two tertiary centers between 2019 and 2024. We extracted clinical demographic variables, biomarker results, and survival times to all-cause-mortality or last seen from electronic health records. SNAP patients were defined based on normal cerebrospinal fluid (CSF) levels of amyloid-β_1-42_ (Aβ_42_) and a normal Aβ_42_/Aβ_40_ ratio, and abnormal levels of phospho-Tau-181 (pTau) and/or total Tau (tTau), as measured with the Lumipulse G chemiluminescence assay. We computed the Youden index to dichotomize SNAP subgroups by CSF biomarkers and demographic variables in a data-driven approach and assessed survival using Kaplan–Meier curves and multivariable Cox hazard models. Patients were categorized as having either idiopathic or secondary neurodegenerative diseases, while those with prion disease were excluded from the final analyses.

**Results:**

Compared across amyloid-tau-neurodegeneration-defined categories, patients with SNAP had a shorter median survival than those with AD (31 vs. 42 months), while the Alzheimer’s pathological change with concurrent neurodegeneration (APC + N) category exhibited the shortest median survival at 16 months. Patients with SNAP (*n* = 99) had a median age of 72 (inter-quartile range, IQR: 64; 77) years and a median follow-up time of 12 (IQR 1;27, max. 60) months. Among selected variables, tTau performed best in predicting survival during the follow-up period (area under the curve, AUC = 0.83) in non-prion SNAP, followed by the pTau/tTau ratio (AUC = 0.82). The optimal prognostic tTau cutpoint of ≥ 600 pg/mL was higher than the age-adjusted diagnostic reference (300—500 pg/mL). Patients with SNAP exhibiting higher tTau levels or a reduced pTau-181/tTau ratio had significantly shorter median survival times. Multivariable Cox hazard modeling confirmed tTau to be independently associated with survival with a hazard ratio of 6.1—6.8 (*p* < 0.001).

**Conclusion:**

We have identified CSF tTau as a novel predictor of survival in patients with SNAP. Although this descriptive diagnosis encompasses primarily various idiopathic neurodegenerative causes, we also demonstrate tTau’s prognostic value in the context of secondary neurodegenerative processes. This finding can enhance prognostication in clinical settings.

**Supplementary Information:**

The online version contains supplementary material available at https://doi.org/10.1007/s00415-026-13841-x.

## Introduction

“Suspected non-Alzheimer pathophysiology” (SNAP) is a biomarker-based diagnostic construct that was introduced with the National Institute on Aging–Alzheimer’s Association recommendations for preclinical Alzheimer’s disease (AD) and their revisions [1–3]. The 2018 framework captures patients without evidence of amyloidosis (A) but with concomitant abnormal biomarkers for tauopathy (T) and/or neurodegeneration (N), contributing to three separate ATN profiles, namely A-T+N-, A-T+N+, and A-T-N+. Biomarker profiles can be fluid- or neuroimaging-based or both. The SNAP concept comprises a spectrum of neurodegenerative entities and hence confers highly variable clinical outcomes [4]. Approximately 16% of patients with mild cognitive impairment and 10% of those with dementia exhibit a diagnostic biomarker profile consistent with SNAP [5–7]. In longitudinal studies of memory clinic populations, cognitive trajectories of patients with SNAP remain relatively stable compared to amyloid-positive patients [8–11]. However, the prognostic implications of cerebrospinal fluid (CSF) AD biomarkers for survival in SNAP, especially beyond classical cognitive impaired syndromes, remain unexplored.

Two autopsy-validated studies confirmed the range of neuropathological entities underlying SNAP, with major contributors being primary age-related tauopathy (17-38%), other primary tauopathies (4-36%), TDP-43-associated disease (18-21%) and even AD neuropathologic change (7-9%) [12, 13]. As a limitation, both studies based their N staging on neuroimaging abnormalities according to the 2011 framework [1] and did not yet include differentiated T and N biomarker groups [2]. A higher prevalence of APOEε2 genotypes than in AD and potential cerebrovascular etiological contributions have been described for SNAP [4, 14].

In previous longitudinal cohort studies in memory clinic settings, patients with SNAP exhibited slower rates of cognitive decline, hippocampal atrophy and Aβ accumulation than A+ patients, with trends more comparable to those observed in A-T-N- patients [8, 10, 11, 15]. At a more granular level, A-T+N- and A-T+N+ profiles supposedly reflect underlying tau pathophysiology, partially independent of amyloid pathophysiology [4]. In contrast, the A-T-N+ profile suggests neurodegeneration independent of both amyloid and tau pathophysiology. Compared to patients with A-T-N- or A-T+N-, those with A-T-N + or A-T+N+ were cognitively more severely impaired and had higher hippocampal atrophy rates [11]. The A-T+N+ profile, however, was associated with an even more rapid clinical progression.

In a broader context, tau biomarkers have been studied for prognostication across diverse diseases [16]. For instance, patients with AD and higher levels of total Tau (tTau) in the CSF exhibit a faster clinical progression and higher mortality rates than those below the reference cut-offs [17]; yet, a recent meta-analysis did not confirm an association of tTau with survival [18]. Similarly, increased CSF tTau was associated with all-cause mortality in patients with dementia with Lewy bodies, amyotrophic lateral sclerosis, and in aged individuals over 85 [19–21].

Despite the extension of the SNAP concept to younger ages and to broader phenotypes beyond AD-type dementia over the years [4], its application in clinical practice and implications for survival outcomes remains incompletely understood. To address this gap, we established a SNAP cohort from two tertiary care centers in Switzerland representing a joint memory clinic and general neurology clinic patient population within which CSF workup was conducted in suspected neurodegenerative disease. From retrospective clinical follow-up, we collected information on CSF biomarkers and clinic-demographic variables to assess survival times in stratified SNAP subgroups.

## Materials and methods

### Patient selection and sampling

Patients were recruited at the Department of Neurology, University Hospital Zurich (USZ) and the Cantonal Hospital St. Gallen (KSSG), from May 2019 to August 2024. Patients at the USZ were recruited from the neurology ward and outpatient clinics for cognitive neurology, movement disorders, emergency neurology, neuromuscular neurology, and neuroimmunology. In contrast, patients at the KSSG were recruited from the outpatient memory clinic. All patients underwent lumbar puncture (LP) and CSF AD biomarker workup in the laboratories at USZ and KSSG. Procedure-specific informed consent was a prerequisite for LP; in non-conscious patients, it was granted by representative relatives or presumed by mutual patient will (e.g., on intensive care units).

## CSF analysis

### CSF AD biomarker workup and ATN operationalization

Internal laboratory protocol standards for evaluation of CSF cytology, chemistry, blood–CSF barrier function and humoral immune response are described elsewhere [22, 23]. Briefly, CSF was processed within 1 to 2 h after LP. White blood cell count and cytology (if cell count ≥ 5 cells/µL) in CSF, as well as albumin, IgG, IgM, and IgA levels in CSF and serum, were quantified, to exclude immunological differential diagnoses.

AD biomarkers were measured in CSF using the Fujirebio Lumipulse® G600II system. Reference ranges were applied according to the vendor’s technical information. Assay reliability is monitored by the Alzheimer’s Association QC program [24]. Cut-offs were defined as follows: Aβ₄₂ 500 pg/mL, Aβ₄₂/Aβ₄₀ (Aβ ratio) 0.0069, pTau-181 56.4 pg/mL and age-adjusted for tTau: 300 pg/mL (< 51 years), 450 pg/mL (51-70 years) or 500 pg/mL (> 70 years).

**Fig. 1:**
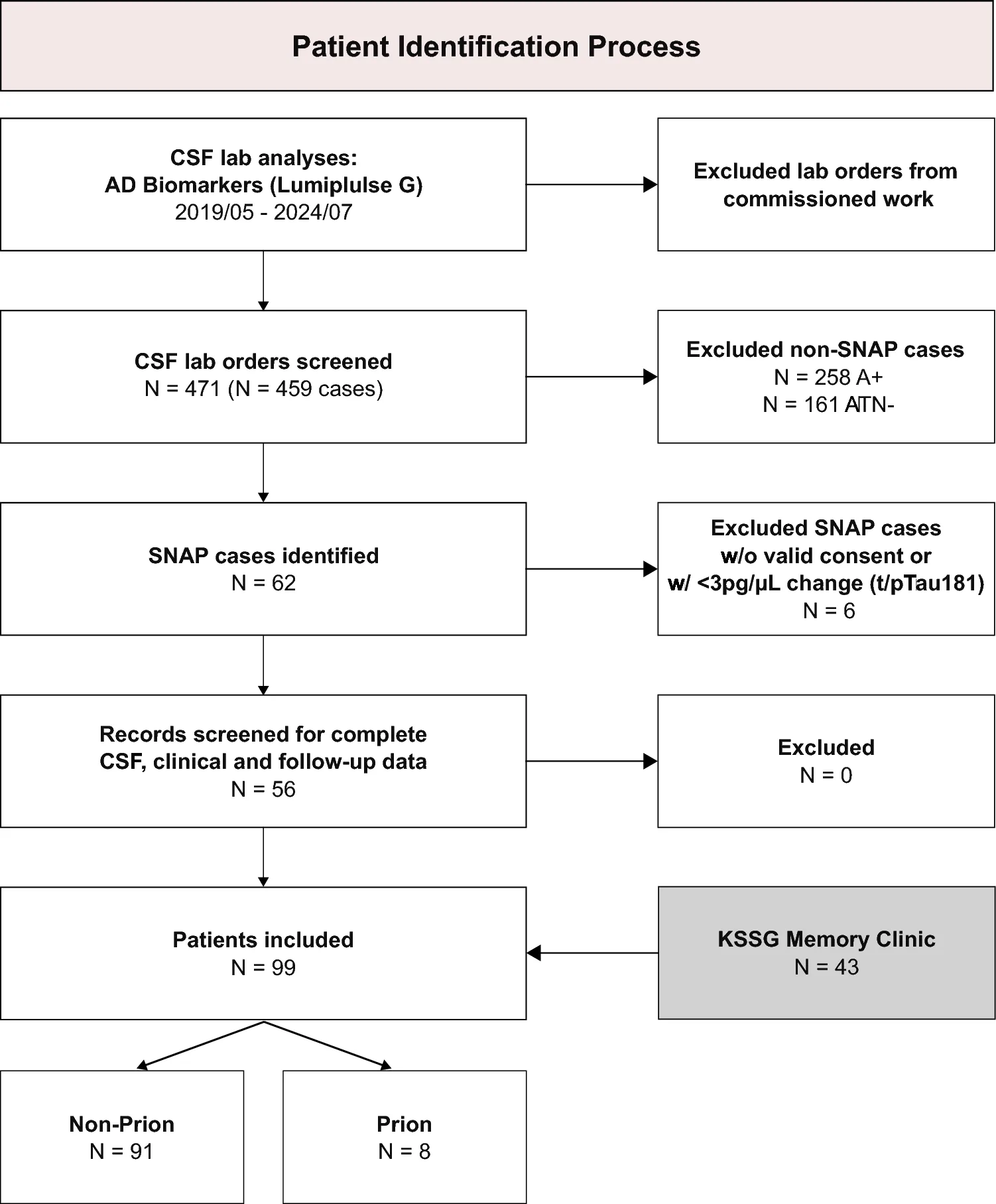
Process of patient identification

We operationalized the ATN framework [2] to classify patients as positive for Aβ pathophysiology (A), tau pathophysiology (T) or neurodegeneration (N) based on measurements of both the Aβ ratio and Aβ₄₂, pTau₁₈₁ or tTau, respectively. N positivity was defined solely by abnormal CSF tTau levels, and no neuroimaging markers were used for ATN operationalization in this study because they were not available for all cases. ATN profiles were further collapsed into ATN categories that comprised normal (NOR; A-T-N-), AD (A+T+N±), Alzheimer’s pathological change (APC; A+T-N-), APC with neurodegeneration (APC+N; A+T-N+) and SNAP (A-T+N-, A-T-N+, A-T+N+). A+ was defined by abnormal absolute Aβ42 and/or abnormal Aβ ratio. Consequently, SNAP patients had Aβ ratio values and Aβ42 concentrations above reference thresholds, as well as pathologically increased pTau₁₈₁ and/or tTau. Cases with borderline values variations around the predefined tTau or pTau-181 cutpoints of < 3 pg/mL were excluded (See Fig. 1).

### Data extraction

Clinical data were extracted by conducting an electronic health record review. We collected data on clinical and postmortem diagnosis (where available), sex, age at LP, disease duration at LP, cognition scores, CSF AD biomarker concentrations, follow-up periods from LP, and survival outcomes.

Cognition was assessed using the Montreal Cognitive Assessment (MoCA), within a time interval of maximum 6 months from LP. We determined the latest contact with a patient by physical or electronic means as documented in the EHR as “last seen” time point. A registered death note was defined as “deceased”, otherwise as “last seen”. The diagnosis was defined by an individualized diagnostic process in routine medical care, integrating neurological-clinical evaluation together with neuropsychological and/or CSF (i.e., CSF AD biomarkers pleocytosis, intrathecal immunoglobulin synthesis) and/or hematological–serological workup and/or neuroimaging.

Over a period of 6 years, 471 CSF AD biomarker lab orders from 459 patients at the USZ site were screened. 258 A+ and 161 A-T-N- analyses were excluded as non-SNAP. After further exclusion of patients without consent or with borderline values, as well as after screening electronic health record for complete CSF workup and clinical data, 56 eligible SNAP patients were identified. To increase the sample size of the SNAP sub-cohort, additional 43 SNAP patients from the KSSG Memory Clinic were integrated. From the combined cohort of both clinics, eight patients were later diagnosed with prion disease, confirmed by a positive prion protein-specific real-time quaking-induced conversion assay from the CSF [25] (RT-QuIC, 8/8 patients, i.e., probable sporadic Creutzfeldt–Jakob disease [CJD], according to Ref. [26]) and/or by autopsy (2/8 patients, i.e., definite sporadic CJD).

### Statistical analysis

Statistical analyses were performed using R 4.5.1 in 2025.09.2 Build 418 for MacOS. Descriptive statistics of cohort characteristics included the calculation of means, medians, and standard deviations for continuous variables, and the calculation of frequencies and percentages for categorical variables. tTau values were log-transformed to approximate a normal distribution. Patient group dichotomization was defined by tTau, pTau-181 levels, pTau-181/tTau ratio, Aβ_42_/Aβ_40_ ratio or age at LP. Optimal cutpoints (oCP) for these variables were determined by maximizing the Youden index with binary survival outcome as target variable, as implemented in the cutpointr package [27]. We compared several classification metrics to predict mortality during the follow-up period, including sensitivity, specificity, accuracy, and area under the curve (AUC). This procedure was conducted for both, the SNAP cohort with and without prion patients to determine their impact on reported oCP. Missing data were addressed using complete case analysis. Inferential statistical tests were chosen based on data type, group balance, data distribution and heteroscedasticity, where applicable, and are indicated in each section. We accepted a p-value ≤ 0.05 to reject the null hypothesis. All source code for this study is available at https://github.com/nes-b/SNAP_svl.

### Survival analysis

The time origin was defined as the date of LP, and patients were followed for a maximum of 60 months or until death. The primary event of interest was time to death. Patients were censored at the date of their last follow-up if they survived the full observation period. We initially conducted univariate analyses with potential predictors preselected by clinical relevance and data completeness, including tTau, pTau-181, Aβ42/Aβ40 ratio, age, and study site. We used the survminer package [28] to create Kaplan–Meier curves and to compute log-rank tests.

We established a Cox proportional hazards model to assess the relationship between predictors and time-to-event outcomes. Model assumptions, specifically proportionality of hazards, were tested by computing scaled Schoenfeld residuals. To maintain adequate statistical power (recommended events per variable, EPV > 6), we fitted a parsimonious multivariable Cox proportional hazards model including tTau group, pTau-181 group, and study site. Given that age demonstrated time-varying effects in proportional hazards testing (Schoenfeld test *p* = 0.03), our primary model was stratified by age group (≥ 75 vs < 75 years) to allow baseline hazards to differ between age strata:

Where h(t|X) represents the hazard function at time t given covariates X, h₀(t|Age) is the baseline hazard function stratified by age group, and β₁, β₂, β₃ are the regression coefficients. A sensitivity analysis including age as an adjusted covariate yielded consistent results (Supplementary Information [SI] 2.6).

Variable selection for the multivariable model was guided by theoretical importance and statistical considerations. We included tTau (primary predictor), pTau-181 (biologically related tau biomarker), and study site (design variable). With 24 mortality events, this yielded an EPV of 8 in the age-stratified model, exceeding recommended thresholds for stable coefficient estimation. Aβ42/Aβ40 ratio was not included as all patients were A-negative by definition (SNAP criteria), and single-variable analysis had not shown a positive signal. Sex and disease duration were excluded to maintain model parsimony given the limited number of events.

An exploratory full model including all covariates (tTau, pTau-181, Aβ42/Aβ40 ratio, age, sex, disease duration, and site; EPV = 3.4, SI 2.6) was additionally fitted to assess robustness of findings, though results should be interpreted with caution given the low EPV. Cox models were specified with the survival package and visualized using survminer and forestmodel packages.

## Results

### Survival stratification by CSF biomarker profiles reveals prognostic heterogeneity

**Fig. 2:**
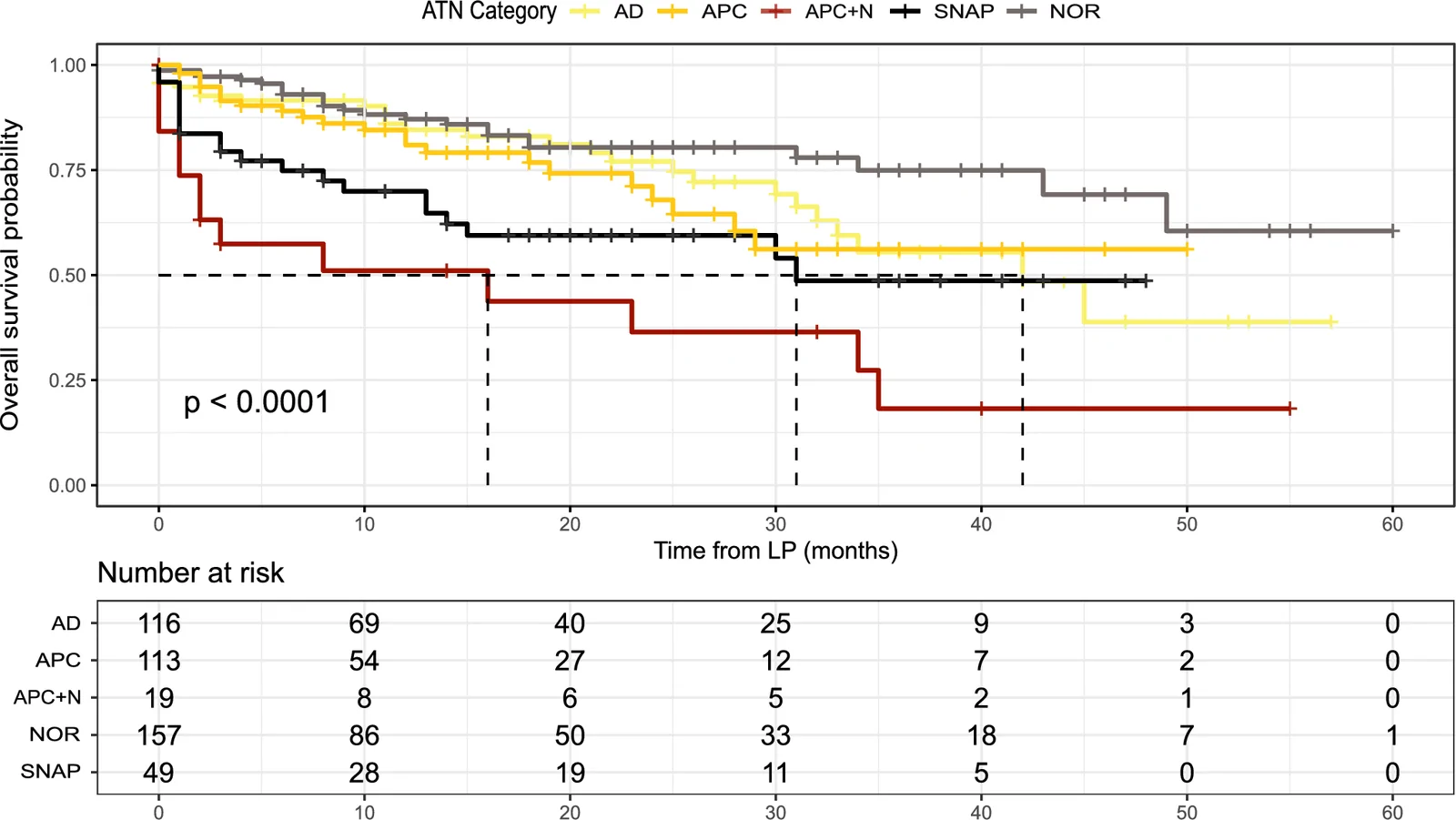
Kaplan–Meier survival curves stratified by CSF biomarker categories. Survival probability is plotted as a function of time elapsed since LP (months). Patients were stratified according to ATN categories (AD, APC, APC+N, NOR; SNAP). The number at risk is displayed below the graph for each time interval. Log-rank test *p* < 0.0001 indicates significant differences in survival distributions across biomarker groups. *AD* Alzheimer’s disease, *APC* Alzheimer’s pathological change, *APC+N* Alzheimer’s pathological change with neurodegeneration, *LP* lumbar puncture, *NOR* normal, *SNAP* suspected non-Alzheimer pathophysiology

To investigate the prognostic value of ATN biomarkers, we conducted survival analyses across different strata, including the CSF-based ATN categories and clinical diagnoses. Patients with neurodegenerative diagnoses exhibited markedly heterogeneous survival trajectories across ATN-defined categories (Fig. 2), but also when stratified by more granular ATN status and clinical diagnoses (SI 2.1, SI 2.2). While individuals with a normal biomarker profile (NOR; A-T-N-) did not reach median survival during the observation period, those with a neurochemical profile of AD (A+T+N±) exhibited a significantly reduced median survival of 42 months (log-rank *p* < 0.0001).

Of particular interest, two distinct subgroups classified as SNAP (A-T+N±, A-T±N+; median survival 31 months) and APC+N (A+T-N+; median survival 16 months) had even lower survival probabilities. The specific diagnoses within these two subgroups are provided in SI1.1. Given the diversity of etiologies underlying SNAP and its potential therapeutic implications, subsequent analyses focused on characterizing prognostic features in SNAP.

### SNAP cohort description

The full SNAP cohort consisted of 99 patients: 56 from USZ with extended follow-up (49 plus 7 new cases) and an additional 43 outpatient memory clinic cases from KSSG. The cohort comprised 57 (58%) males, with a median age at LP of 72 (IQR: 64-77) years, a median follow-up time of 12 (IQR: 1-27) months, and a median MoCA score of 20.0 (IQR: 17.3-25.0) points (Table 1). Patients who died during follow-up had shorter disease duration prior to CSF sampling (< 1 vs. 1 year, *p* = 0.031) and higher absolute tTau levels (915 vs. 515 pg/mL, *p* < 0.001), a lower pTau-181/tTau ratio (0.05 vs. 0.12, *p* < 0.001), and a lower Aβ_42_/pTau-181 ratio (22 vs. 31, *p* = 0.003) (Fig. 2). Absolute Aβ_42_ and Aβ_40_ levels were lower in patients with SNAP who died within 1 year (both *p* < 0.01); however, the Aβ_42_/Aβ_40_ ratio was unchanged.

**Table 1:** Cohort characteristics

			Mortality outcome			ATN			tTau group		
Variable	*N*	Overall *N* = 99^*1*^	Survived *N* = 75^*1*^	Deceased *N* = 24^*1*^	*p*^*2*^	A-T-N + *N* = 54^*1*^	A-T+N ± *N* = 45^*1*^	*p*^*2*^	Low *N* = 62^*1*^	High *N* = 37^*1*^	*p*^*2*^
Age at LP [y]	98	72 (64, 77)	73 (64, 78)	71 (66, 74)	0.2	70 (61, 76)	75 (69, 78)	**0.004**	74 (67, 78)	70 (63, 75)	*0.074*
Sex [M]	99	57 (58%)	43 (57%)	14 (58%)	> 0.9	32 (59%)	25 (56%)	0.7	38 (61%)	19 (51%)	0.3
Disease duration at LP [y]	56	0.00 (0.00, 2.25)	1.00 (0.00, 3.00)	0.00 (0.00, 1.00)	**0.031**	0.00 (0.00, 1.00)	1.00 (0.00, 3.00)	**0.013**	1.00 (0.00, 3.00)	0.00 (0.00, 0.75)	**0.002**
FU time from LP [m]	96	12 (1, 27)	18 (1, 29)	4 (1, 11)	**0.043**	14 (1, 27)	8 (1, 23)	0.6	18 (1, 30)	3 (1, 17)	0.14
Mortality status	99	24 (24%)				15 (28%)	9 (20%)	0.4	4 (6.5%)	20 (54%)	** < 0.001**
MoCA	74	20.0 (17.3, 25.0)	20.0 (18.0, 25.0)	20.0 (13.0, 25.0)	> 0.9	21.0 (18.8, 25.0)	19.0 (16.0, 23.8)	**0.048**	20.0 (17.0, 25.0)	20.0 (18.0, 25.0)	0.6
Prion etiology	99	8 (8.1%)	0 (0%)	8 (33%)	** < 0.001**	5 (9.3%)	3 (6.7%)	0.7	0 (0%)	8 (22%)	** < 0.001**
Biomarkers											
tTau [pg/mL]	99	533 (467, 785)	515 (459, 576)	915 (657, 2273)	** < 0.001**	520 (458, 932)	540 (500, 659)	0.6	485 (442, 526)	962 (768, 1845)	** < 0.001**
pTau-181 [pg/mL]	99	55 (40, 65)	56 (40, 65)	52 (44, 70)	0.7	44 (31, 51)	68 (61, 76)	** < 0.001**	59 (48, 65)	50 (37, 68)	0.2
pTau-181/tTau	99	0.10 (0.05, 0.12)	0.12 (0.07, 0.13)	0.05 (0.02, 0.06)	** < 0.001**	0.07 (0.04, 0.10)	0.12 (0.11, 0.13)	** < 0.001**	0.12 (0.10, 0.13)	0.04 (0.02, 0.07)	** < 0.001**
Aβ_42_ [pg/mL]	99	1,330 (923, 1,913)	1500 (1,013, 1,951)	959 (883, 1273)	**0.007**	1195 (834, 1638)	1,561 (1178, 1984)	**0.001**	1543 (1,102, 1947)	1,066 (844, 1,500)	**0.01**
Aβ_40_ [pg/mL]	99	13,461 (10,099, 17,681)	14,270 (11,339, 18,635)	10,515 (8,578, 12,555)	**0.003**	11,604 (8526, 14,830)	15,850 (12,374, 19,745)	** < 0.001**	14,617 (11,458, 18,448)	11,018 (8604, 14,550)	**0.008**
Aβ_42_/Aβ_40_	99	0.103 (0.094, 0.111)	0.104 (0.097, 0.111)	0.099 (0.088, 0.109)	0.3	0.105 (0.097, 0.111)	0.101 (0.089, 0.111)	0.2	0.105 (0.096, 0.111)	0.102 (0.089, 0.108)	0.3
Aβ_42_/pTau-181	99	30 (20, 35)	31 (23, 35)	22 (16, 28)	**0.003**	32 (26, 37)	23 (17, 31)	** < 0.001**	30 (23, 35)	27 (19, 34)	0.2

Demographic and clinical characteristics of the SNAP cohort, categorized by mortality outcome, ATN classification, and tTau levels. Statistical tests used are the Wilcoxon rank-sum test (nominal), Pearson’s chi-squared test, and Fisher’s exact test. Significant and trending p-values are bold or italic, respectively. ^1^ Median (IQR); *n* (%). ^2^ Wilcoxon rank-sum test; Pearson’s chi-squared test; Fisher’s exact test. *F* Female, *FU* follow-up time, *LP* lumbar puncture, *MoCA* Montreal Cognitive Assessment, *M* months, *Y* years

All patients with prion disease, diagnosed by CSF PrP RT-QuIC and/or by autopsy, deceased during the follow-up period. Integrated clinical diagnoses of non-prion cases comprised primary neurodegenerative or idiopathic (i.e., frontotemporal dementia, atypical Parkinsonian syndrome, motor neuron disease, and progressive traumatic encephalopathy syndrome), and secondary neurodegenerative etiologies (i.e., vascular dementia, autoimmune/demyelinating CNS conditions, neuro-oncological and epileptic conditions) (SI 1.1).

Differentiation of SNAP profiles according to a CSF-based ATN classification scheme (i.e., normed assay- and laboratory-specific reference ranges, see Methods) resulted in three imbalanced groups with 54 A-T-N +, 2 A-T+N- and 45 A-T+N+ cases. Given the small sample size of the A-T+N- subgroup, its similarity with the A-T+N+ subgroup in clinic-demographic variables, and the typical sequence of conversion from A-T+N- to A-T+N+, we joined these groups together for further comparisons [29]. Notably, while age at LP, disease duration, MoCA scores, absolute Aβ markers and Aβ42/pTau ratio differed significantly, the Aβ ratio and mortality rates did not.

### tTau and pTau-181/tTau predict mortality in SNAP

**Fig. 3:**
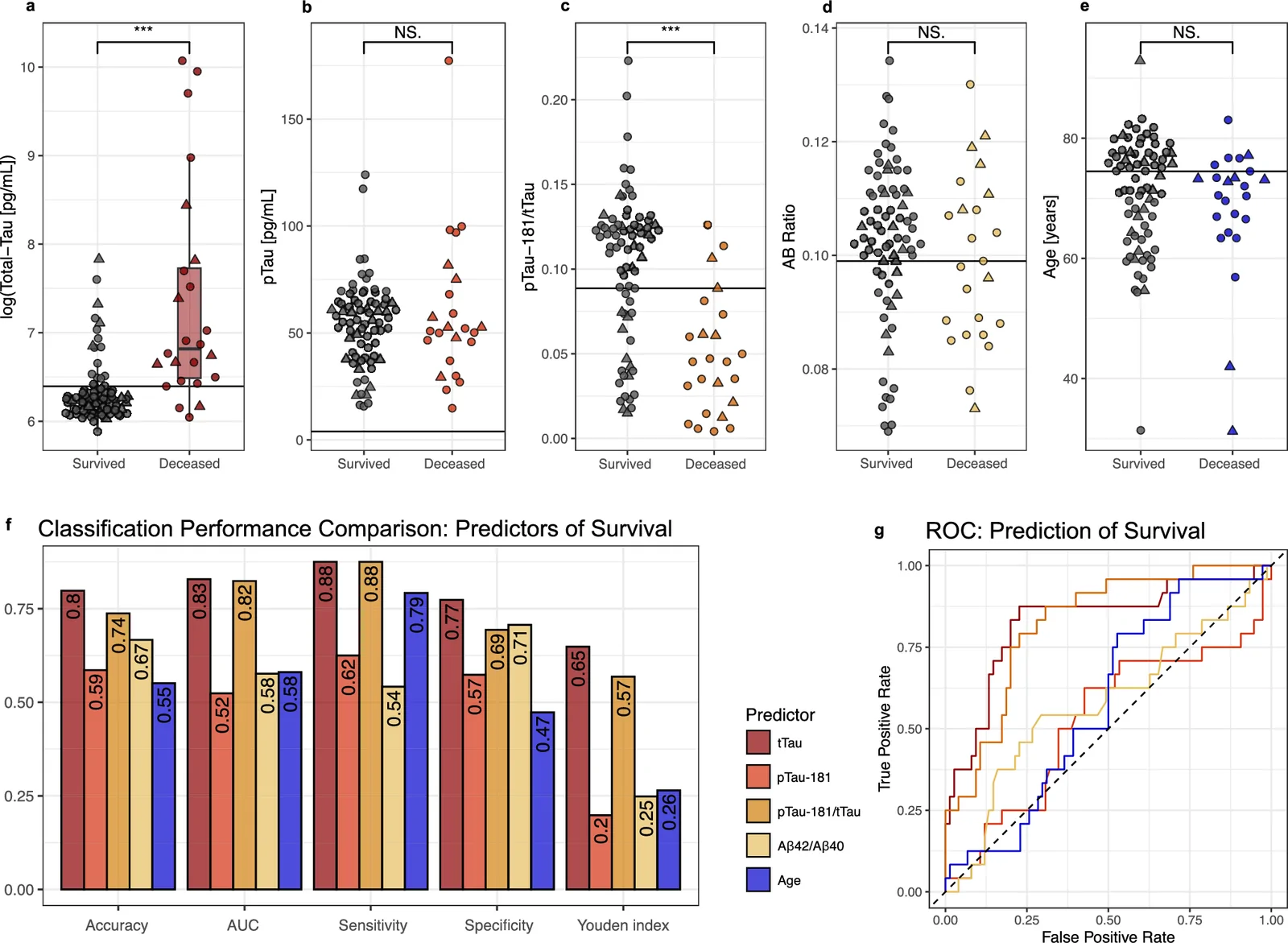
Biomarker analysis for mortality prediction. **a–e** Distribution of tTau (log-transformed), pTau, pTau-181/tTau ratio, Aβ ratio, and age, in survivors vs. deceased patients with SNAP. Shape depicts primary (circle) or secondary (triangle) neurodegenerative etiology. Wilcoxon rank-sum tests, ***p = 0.001, NS (not significant). **f** Comparison of classification performance metrics (accuracy, AUC, sensitivity, specificity, Youden index) for survival prediction using tTau, pTau, pTau-181/tTau ratio, Aβ ratio, and age. **g** *ROC* Receiver operating characteristic curves for survival prediction for survival prediction using tTau, pTau, pTau-181/tTau ratio, Aβ ratio, and age

We conducted a data-driven approach to dichotomize continuous biomarker levels to identify individuals at high mortality risk. We computed the Youden index to identify optimal cutpoints (oCP) for continuous variables CSF tTau, pTau-181, pTau-181/tTau and Aβ_42_/Aβ_40_ ratios as well as age at LP (SI 1.2 SI 1.3, SI 2.3). We compared potential prognostic biomarkers and variables by their prediction performance of mortality during the follow-up period. Among these, tTau performed best in discriminating SNAP patients who survived against those who deceased (oCP = 599 pg/mL, AUC = 0.83, sensitivity = 88%, specificity = 77%). This cutpoint was higher than our age-adjusted reference of 300-500 pg/mL, and absolute CSF levels exceeded those of survivors by up to 2 logs (Fig. 3). The pTau-181/tTau ratio was reduced in deceased SNAP and predicted the mortality state at comparable sensitivity, albeit with lower specificity (oCP = 0.089, AUC = 0.82, sensitivity 88%, specificity = 69%). Remaining variables at test, including age (oCP = 75 years, AUC = 0.58), Aβ_42_/Aβ_40_ (oCP = 0.099, AUC = 0.58) and pTau (oCP = 58.6 pg/mL, AUC = 0.52) were less suitable predictors.

When dichotomizing the SNAP cohort by the tTau oCP, resulting tTau^HIGH^ and tTau^LOW^ groups were of comparable ages (*p* = 0.074) and sex distribution (*p* = 0.3). However, these groups differed with regard to disease duration (< 1 vs. 1 year, *p* = 0.002), frequency of prion etiology (22 vs. 0%, *p* < 0.001) and mortality outcome (54 vs. 6.5%, *p* < 0.001). We further observed negative correlations of tTau levels with disease duration (rho = −0.35, *p* = 0.015, SI 2.4) in non-prion cases and with follow-up times in prion cases (rho = −0.78, *p* = 0.028), but without any significant relation to MoCA scores. While broad diagnostic categories were not preferentially distributed within either mortality stratum (Fig. 3a, point shape), several specific diagnoses, such as prion disease, new-onset refractory status epilepticus, and stiff person spectrum disease, were found among deceased patients and exhibited the highest tTau levels (SI 2.5). Prion disease is a well-established cause of very high CSF tTau levels, and the prognosis after symptom onset is poor, with a median survival of 5 months [30]. Therefore, we excluded patients with prion diseases from further downstream analyses. We retained a single motor neuron disease (MND) patient in the non-prion SNAP cohort, as MND does not cause tTau elevations comparable to those seen in prion disease, making it less likely to confound the tTau survival analysis [31].

To assess whether this more conservative SNAP definition influences the cutpoints, we repeated the oCP calculation in the SNAP cohort after excluding these prion patients (SI 1.2). Thereby, we confirmed a similar tTau oCP of 599 pg/mL, however, with slightly lower sensitivity (0.813 vs. 0.875). The pTau-181 oCP increased modestly from 51 to 59 pg/mL. Overall, the prognostic performance of the oCPs remained comparable to the prion-inclusive analysis.

### tTau as independent predictor of survival in non-prion SNAP

**Fig. 4:**
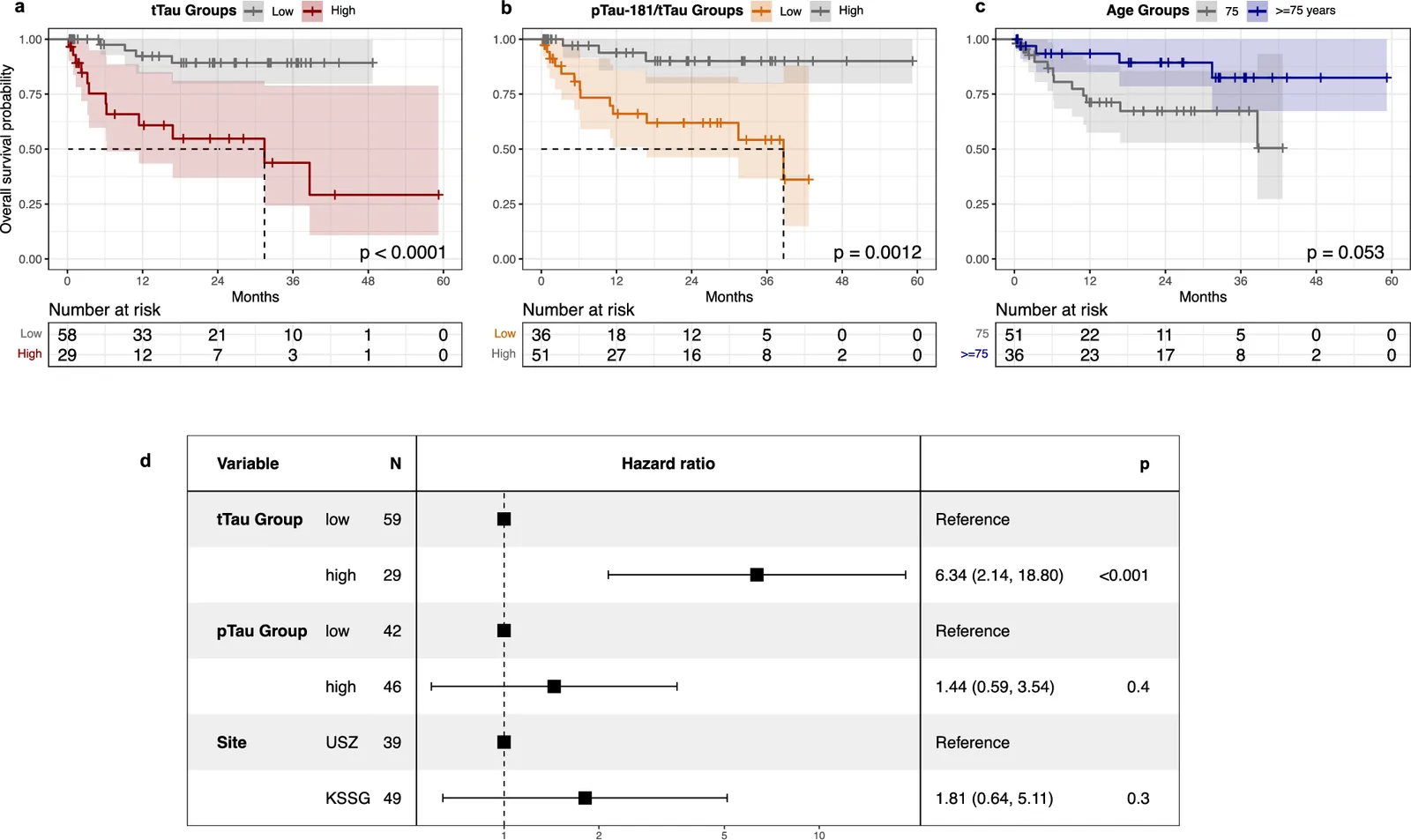
Survival analysis for mortality prediction. **a–c** Kaplan–Meier curves of event-free survival for patients with SNAP divided into tTau, pTau-181/tTau ratio, and age groups. *P*-values from log-rank tests. Lower panels indicate numbers at risk. **d** Results from multivariable Cox proportional hazard model for biomarker groups and recruiting site. Left column indicates variables, their values and number of available data points in the left column. Middle column indicates forest plot of hazard ratios with mean (box) and confidence interval (whiskers). Right column indicates raw values of hazard ratios and confidence intervals, and *p*-values

Next, we conducted time-dependent survival analyses employing event-free follow-up durations until death or last seen, with biomarkers and clinic-demographic variables serving as predictors. In comparison to the tTau_LOW_ group, the tTau_HIGH_ group exhibited a significantly reduced median survival (31 months; log-rank *p* < 0.0001, Fig. 4a). Additionally, the pTau-181/tTau ratio demonstrated prognostic significance (*p* = 0.0012, Fig. 4b). Notably, a younger age in SNAP trended towards higher mortality (*p* = 0.053, Fig. 4c).

To adjust for confounding effects of multiple biologically related predictors on mortality, we established staged multivariable Cox proportional hazard models, incorporating tTau, pTau-181, and recruiting site, as well as Aβ ratio, age (≥ 75 vs. < 75), sex, and etiology types. Thereby, we confirmed tTau (HR = 6.70, 95% CI 2.08–21.56, *p* = 0.001, Fig. 4d, SI 2.7) as an independent predictor of mortality in patients with SNAP. Of note, there was a concentration-dependent relation between increased tTau levels and worse outcomes in SNAP, with and without including prion cases (SI 2.6). The Aβ ratio (*p* = 0.598) and pTau-181 (*p* = 0.398) groups, age < 75 years (*p* = 0.113), sex (*p* = 0.683), site (*p* = 0.251) and etiology types (primary/idiopathic vs. secondary, *p* = 0.6) were not prognostically relevant (SI 2.7).

## Discussion

Our study provides novel insights into the prognostic value of CSF biomarkers for survival in SNAP. CSF tTau levels emerged as the strongest predictor of mortality among the variables examined. A data-driven cutpoint estimate of ≥ 600 pg/mL for tTau effectively stratified patients with SNAP into high- and low-risk groups for mortality, after adjusting for other biomarkers and clinical variables. Additionally, younger age and longer disease duration were all associated with higher mortality risk, albeit with markedly lower predictive performance (AUC < 0.6 for both, vs. 0.83 for tTau) and smaller hazard ratios in the multivariable model.

This study contributes to our understanding of SNAP and its clinical implications. First, as our cohort comprises a broad clinical and etiological spectrum including patients with mild cognitive impairment and dementia with both primary neurodegenerative and secondary neurodestructive underlying processes, we demonstrate the clinical usefulness of the SNAP biomarker category and tTau as a unifying prognostic marker. However, as the stratification according to a modified higher CSF tTau cutpoint indicates, the recognition of the more granular ATN biomarker profiles (i.e., A-T-N+ or A-T+N+) is essential for a more precise prognostication.

Second, given the etiological diversity within the tTau_HIGH_ SNAP group, converging pathways of neurodegeneration may influence survival outcomes. From a causality perspective, it is most reasonable to view the exceeding tTau levels as expression of a rapidly progressive neurodegenerative process, occurring either as a primary neurodegenerative or as secondary phenomenon to a non-degenerative disease. The neurodestructive process in turn mediates the actual mortality by affecting essential networks for e.g., respiration or consciousness. CSF tTau may be less sensitive to detect neuronal damage and consequently predict mortality than neurofilament light chain, both of which rise in primary neurodegenerative disease, cerebrovascular disease, epileptic seizures, and traumatic brain injury [29, 32].

While CSF tTau levels correlate with disease severity and consequently rise in later stages of AD, its longitudinal dynamics in the SNAP context are largely unknown [29]. In neurodegenerative contexts such as dominantly inherited AD, CSF tTau estimated annual rates change slowly over several years [33]. This slow CSF tTau increase appears to rely on amyloid positivity, at least when studied along the sporadic AD continuum [34]. In contrast, in acute conditions such as traumatic brain injury, CSF tTau levels peak 4.5 to 5.1 days after the impact [35].

Higher CSF tTau levels in the SNAP tTau_HIGH_ group can theoretically result from various mechanisms: increased neuronal production and/or secretion (e.g., from epileptic seizure activity, or excitotoxicity), increased transfer to the CSF from brain parenchyma (e.g., from ependymal barrier dysfunction), or reduced tTau clearance (i.e., from the brain parenchyma, CSF, or plasma) [36]. Given the relatively rapid turnover of tTau in the CSF (i.e., T₁/₂ ~ 9.9 h), chronically elevated levels likely reflect ongoing neurodegenerative processes rather than just accumulated damage [36]. On the contrary, acute conditions that induce short-lived CSF tTau peaks might be overlooked if CSF analysis is performed outside the deviation time window. However, as our study indicates, increased tTau was associated with increased mortality in the scale of months independently of the estimated disease duration until LP, suggesting a long-lived perpetuating process maintaining these tTau levels in the CSF. Nevertheless, whether even short-lived CSF tTau peaks are associated with higher intermediate-term mortality remains to be answered. In clinical practice, a re-puncture could be evaluated to rule out only short-lived CSF tTau increments. Despite these limitations and considerations, the intermediate-term dynamics of CSF tTau with sustained increases over days and strong demonstrated effect sizes in our SNAP cohort support its value as prognostic biomarker for mortality risk.

The prognostic value of CSF tTau in other neurodegenerative conditions has been well established. In clinically more rapidly progressing entities such as prion disease, amyotrophic lateral sclerosis and non-inherited frontotemporal dementia as well as in dementia with Lewy bodies and aged individuals over 85, higher tTau levels have been linked to poorer survival outcomes [19–21, 30]. In traumatic brain injury, CSF tTau was markedly increased in individuals with poor short-term survival times [37]. Similarly, in a stratified cohort study of 151 patients with AD treated with acetylcholine esterase inhibitors, elevated CSF tTau (and reduced Aβ₄₂) predicted higher mortality rates and worse treatment responses [17]. However, a recent meta-analysis assessing biomarker–mortality risk associations across AD cohorts, which did include the aforementioned study, faced problems with inconsistent cutpoints provided and could not confirm an association of tTau with survival [18]. Additionally, in the elderly general population aged over 65, tTau in plasma was not prognostically relevant for all-cause-mortality [38]—a null-finding probably related to worse signal-to-noise ratios of tTau in blood [16].

Nevertheless, the broad consistency of tTau’s prognostic value across various neurodegenerative conditions underscores its utility in clinical practice. However, it is important to note that the relationship between tTau levels and disease progression is nonlinear, with a late-stage increase in tTau levels with consecutive slowing in AD contexts [39, 40]. This complexity stresses the need for careful interpretation of tTau levels in the context of other clinical and biomarker data.

Our findings corroborate CSF tTau as a valuable prognostic tool in SNAP, beyond traditional AD diagnosis and monitoring, and enable risk stratification for clinical decision-making, patient counseling, and research trial design. For clinicians encountering patients with SNAP, tTau levels of ≥ 600 pg/mL should prompt consideration of accelerated disease trajectories and intensified supportive care planning. It should be noted that this 600 pg/mL cutpoint is specific to the Lumipulse G platform; analogous thresholds for other immunoassay systems require independent calibration, given the systematic inter-platform variability in absolute tTau values documented across the field [18]. Notably, the prognostic value of tTau was independent of pTau-181, Aβ42/Aβ40 ratio and of clinical etiology type in the multivariable model, suggesting that the neurodestructive burden reflected by elevated tTau—rather than the underlying proteinopathy—is the principal determinant of survival outcomes within this SNAP cohort.

Future prospective studies should 1) validate the 600 pg/mL cutpoint in independent cohorts of patients with SNAP; 2) investigate whether serial tTau measurements improve prognostic accuracy; 3) examine tTau dynamics in relation to disease-specific interventions; 4) integrate tTau with additional markers such as neurofilament light chain, synaptic proteins, and neuroimaging metrics for multidimensional risk modeling; and 5) explore whether tTau increments vary between specific SNAP subpopulations defined by disease entities.

### Limitations

Several limitations warrant acknowledgment. The relatively small cohort (n = 99) limits statistical power, especially for strata, and generalizability, necessitating validation in larger multicenter studies. The retrospective design introduces potential ascertainment and information biases, particularly regarding the last seen time point. The inclusion of neuroimaging-based neurodegeneration (e.g., hippocampal volumes, cortical thickness) and plasma markers would have allowed comprehensive multimodal staging according to the ATN [2] or the revised ATNIVS criteria [3], though CSF-based operational definitions align with current frameworks. Additionally, the observational setting results in heterogeneous follow-up intervals and variable completeness of clinical data, which may introduce unaccounted confounding.

A conceptual limitation is that tTau contributes to both the definition of the A-T-N+ SNAP subgroup and to the prognostic model. This potential circularity is attenuated by the substantially higher prognostic threshold (600 pg/mL) compared to the diagnostic cutpoint, by the continuous nature of the predictor, and by the inclusion of the A-T+N± subgroup where N positivity is not a required classification criterion. Nevertheless, this design warrants consideration when interpreting the findings.

Importantly, while tTau emerged as the strongest predictor in our cohort, neurofilament light chain (not measured in this study) may capture more general aspects of neurodegeneration in certain contexts and should be incorporated in future investigations. The interpretation of tTau elevation as reflecting ongoing synaptic degeneration, while supported by recent mechanistic studies [41], remains inferential in our dataset and would benefit from validation with synaptic biomarkers and longitudinal tTau trajectories.

## Conclusions

Our study provides compelling evidence for the prognostic value of CSF tTau in patients with SNAP, offering a potential tool for risk stratification in this heterogeneous group. The identification of a data-driven cutpoint of ≥ 600 pg/mL for tTau effectively distinguished high- and low-risk SNAP patients for mortality outcomes. These findings provide a further basis for larger, prospective studies to further elucidate the clinical utility of tTau and other cross-disease biomarkers in managing patients with SNAP. The results suggest that despite the etiological heterogeneity within SNAP, CSF tTau serves as a unifying prognostic marker that can aid clinical decision-making and patient counseling.

## Supplementary Information

Below is the link to the electronic supplementary material.

Supplementary file1 (PDF 2500 KB)

## Data Availability

The datasets used and analyzed during the current study can be made available from the corresponding author upon reasonable request.

## Abbreviations

AD: Alzheimer’s disease
ATN: Amyloid, tau, neurodegeneration
AUC: Area under the curve
CSF: Cerebrospinal fluid
EPV: Events-per-variable
HR: Hazard ratio
IQR: Interquartile range
KSSG: Cantonal Hospital St. Gallen
LP: Lumbar puncture
MoCA: Montreal Cognitive Assessment
oCP: Optimal cutpoint
pTau: Phosphorylated tau
RT-QuIC: Real-time quaking-induced conversion
SNAP: Suspected non-Alzheimer pathophysiology
tTau: Total tau
USZ: University Hospital Zurich
