# Supplementary material for: Outcome associations of CSF total tau in suspected non-Alzheimer pathophysiology
Source: J Neurol. 2026 Apr 30;273(5):296. doi: 10.1007/s00415-026-13841-x (PMC13668912; doi:10.1007/s00415-026-13841-x)
Supplement: Supplementary file 1 [file 415_2026_13841_MOESM1_ESM.pdf]

Supplementary Information (SI) 2

Journal of Neurology

CSF Total-Tau Predicts Survival in Suspected Non-Alzheimer Pathophysiology

Authors:

Nils Briel[1,2], Marc Borsi[1], Simon J. Schreiner[1], Thomas Schneider[3,4], Sandra V. Loosli[1], Claudio Togni[1], Manfredi C. Carta[1], Jan Loosli[1], Alexander Köpp[1,2], Mario Ziegler[1], Tommaso P. Nicoletti[1,5,6], Michael Weller[1], Ansgar Felbecker[3,7], Tobias Weiss[1]*, Hans H. Jung[1]*

**Contributed equally*

1. University Hospital Zurich and University of Zurich, Department of Neurology, Frauenklinikstrasse 26, CH-8091 Zurich, Switzerland
2. Department of Chemistry and Applied Biosciences (D-CHAB), ETH Zurich, Vladimir-Prelog-Weg 1-5/10, CH-8093 Zurich, Switzerland
3. Department of Neurology, HOCH Health Ostschweiz, Rorschacher Strasse 95, CH-9007 St. Gallen, Switzerland
4. Division of Clinical Geriatrics, Department of Neurobiology, Care Sciences and Society, Center for Alzheimer Research, Karolinska Institutet, Huddinge, 141 52, Sweden
5. Department of Neuropathology, Pitié-Salpêtrière Hospital, AP-HP Sorbonne University, Paris, France
6. Institut du Cerveau, Paris Brain Institute, ICM, Inserm U1127, CNRS UMR7225, APHP, Sorbonne University, Pitié-Salpêtrière Hospital, Paris, France
7. Department of Neurology, Inselspital, University Hospital Bern, Rosenbühlgasse 25, CH-3010 Bern, Switzerland

Correspondence

Nils Briel, Dr. med.
Department of Neurology
University Hospital Zurich
Frauenklinikstrasse 26
8091 Zurich, Switzerland
E-mail: nils.briel@usz.ch

Index

SI 1. Supplementary Tables

SI 2. Supplementary Figures

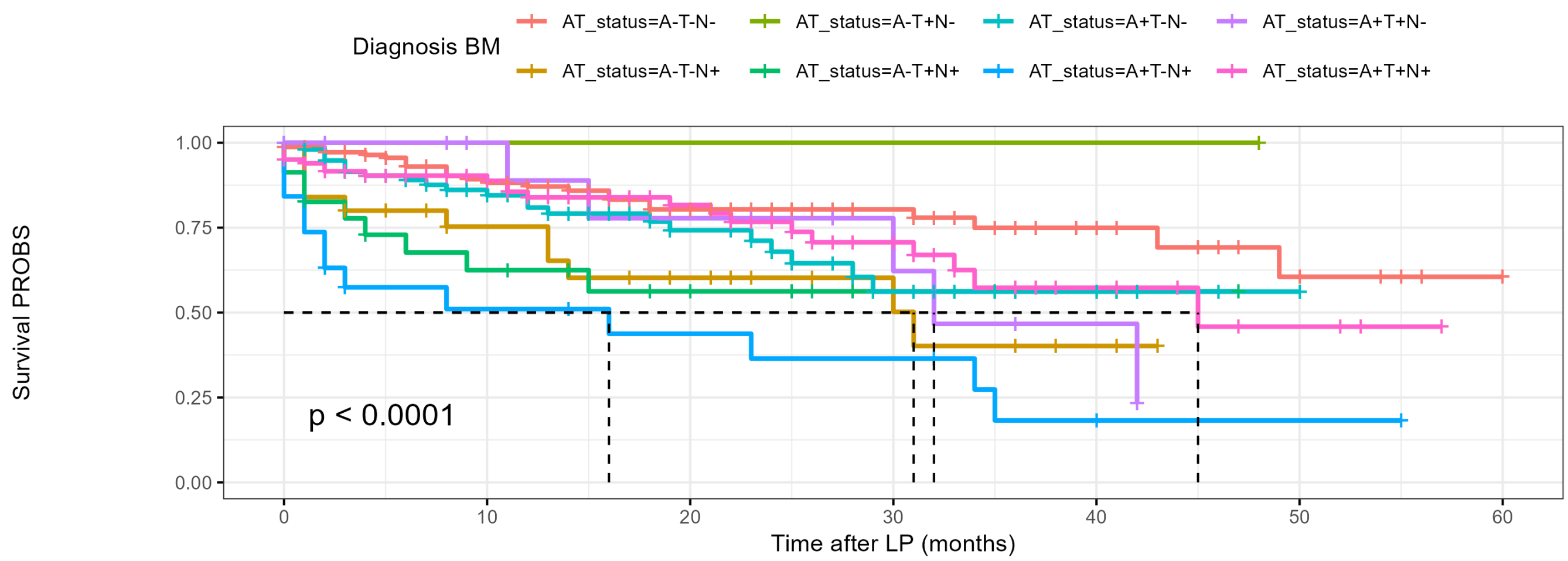

Number at risk

Diagnosis BM	0	10	20	30	40	50	60
AT_status=A-T-N-	157	86	50	33	18	7	1
AT_status=A-T-N+	25	15	10	6	2	0	0
AT_status=A-T+N-	1	1	1	1	1	0	0
AT_status=A-T+N+	23	12	8	4	2	0	0
AT_status=A+T-N-	113	54	27	12	7	2	0
AT_status=A+T-N+	19	8	6	5	2	1	0
AT_status=A+T+N-	15	9	7	5	2	0	0
AT_status=A+T+N+	101	60	33	20	7	3	0

Time after LP (months)

SI 2.1 Survival Analysis of the Entire AD/Non-AD Cohort Stratified by ATN Profiles

Kaplan-Meier curves of event-free survival in patients with SNAP stratified according to the ATN classification system. P-values from log-rank tests. Lower panels indicate numbers at risk.

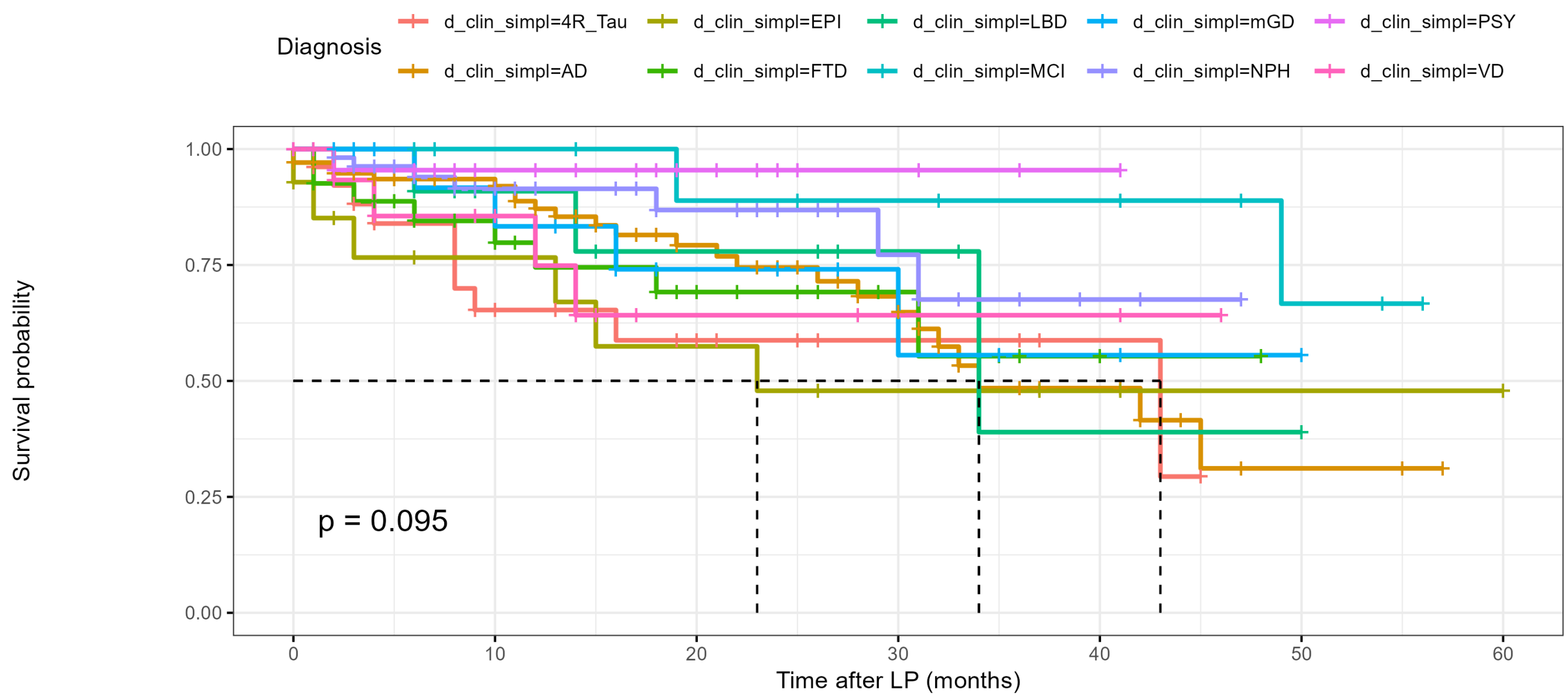

Number at risk

	0	10	20	30	40	50	60
d_clin_simpl=4R_Tau	28	12	8	4	2	0	0
d_clin_simpl=AD	103	61	33	20	8	2	0
d_clin_simpl=EPI	14	8	6	3	2	1	1
d_clin_simpl=FTD	30	18	10	5	2	0	0
d_clin_simpl=LBD	15	7	5	3	1	1	0
d_clin_simpl=MCI	18	10	8	7	6	3	0
d_clin_simpl=mGD	20	11	6	4	2	1	0
d_clin_simpl=NPH	61	31	17	8	2	0	0
d_clin_simpl=PSY	26	16	7	3	1	0	0
d_clin_simpl=VD	19	9	3	2	2	0	0

SI 2.2 Survival Analysis of the Entire AD/Non-AD Cohort Stratified by Clinical Diagnoses

Kaplan-Meier curves of event-free survival in patients with SNAP stratified according to the clinical diagnoses. P-values from log-rank tests. Lower panels indicate numbers at risk.

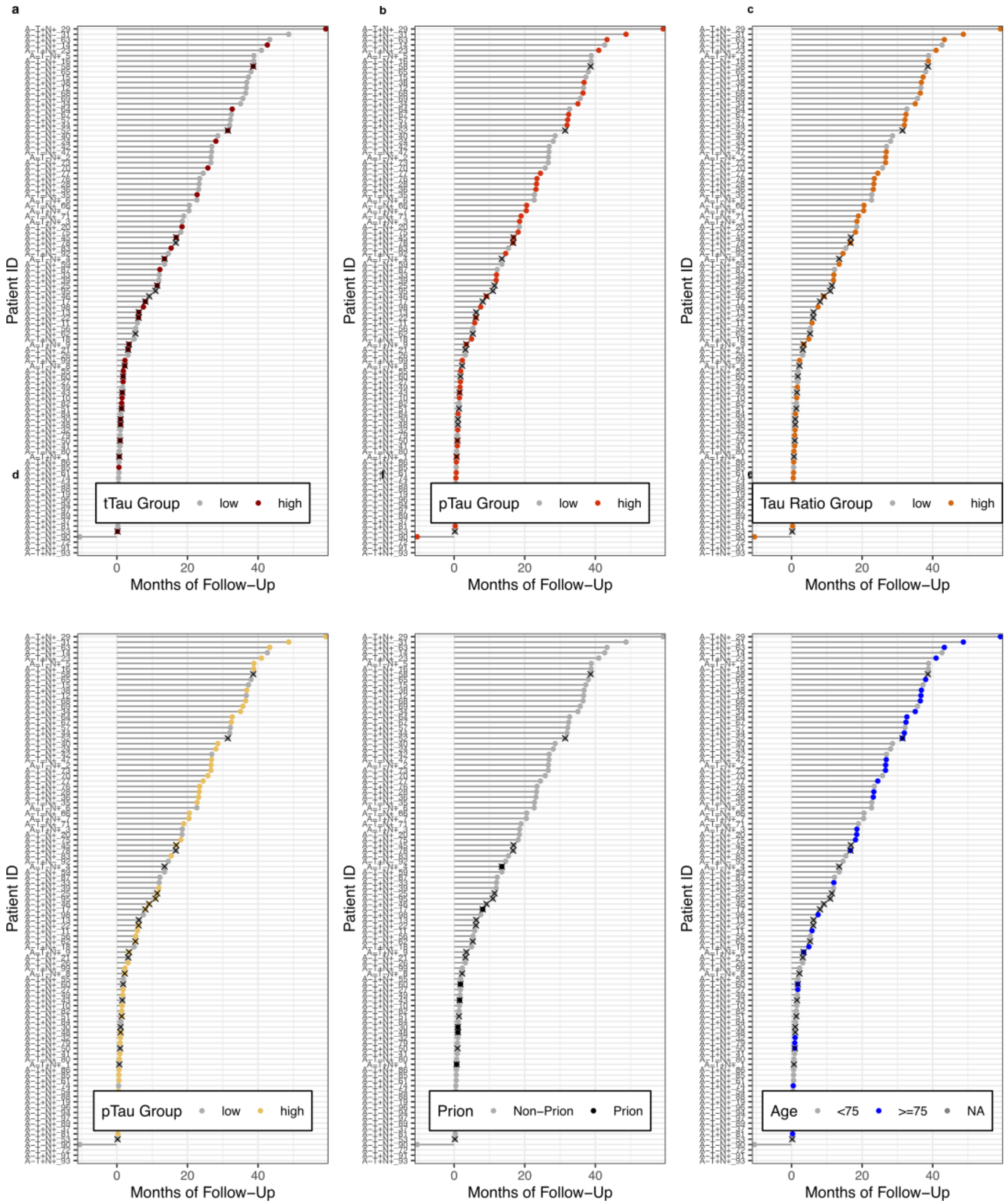

SI 2.3 SNAP Patient Follow-Up Data Categorization

Illustration of individual patient follow-up data categorized by: **a**. tTau group with a cutpoint of 599 pg/mL. **b**. pTau group with a cutpoint of 52.6 pg/mL. **c**. Tau ratio group with a cutpoint of 0.089. **d**. Aβ ratio group with a cutpoint of 0.099. **e**. Prion etiology, distinguishing prion from non-prion conditions. **f**. Age, using a cutpoint of 75 years.

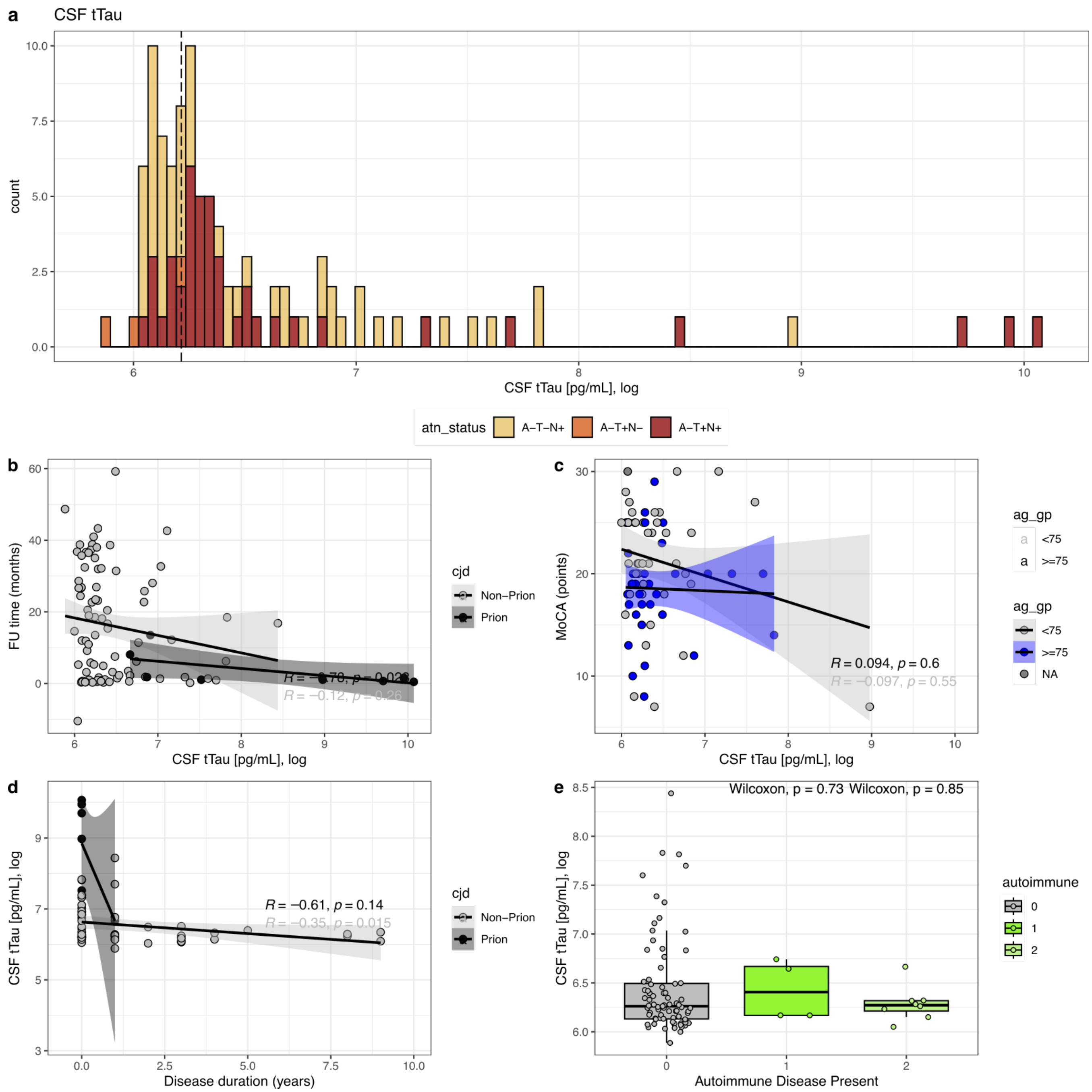

SI 2.4 Relation Of CSF Tau Biomarkers to Clinic-Demographic Variables In SNAP

a. Histogram of CSF tTau levels (log scale) categorized by ATN status (A-T-N-, A-T-N+, A+T-N+). **b**. Scatter plot of follow-up time (FU) against CSF tTau levels (log scale). Prion and non-prion etiology is distinguished. Spearman's Rho and p-value as indicated. **c**. Scatter plot of CSF tTau levels (log scale) against MoCA scores. Age groups (ag_gp, <75 vs. ≥75) are distinguished. Spearman's Rho and p-value as indicated. **d**. Relationship between CSF tTau levels (log scale) and disease duration. Prion and non-prion etiology is distinguished. Spearman's Rho and p-value as indicated. **e**. Box plots comparing CSF tTau levels in SNAP and co-morbid autoimmune disease of the CNS (1) or non-CNS (2) against those without a record of autoimmune disease (0). Wilcoxon rank-sum tests.

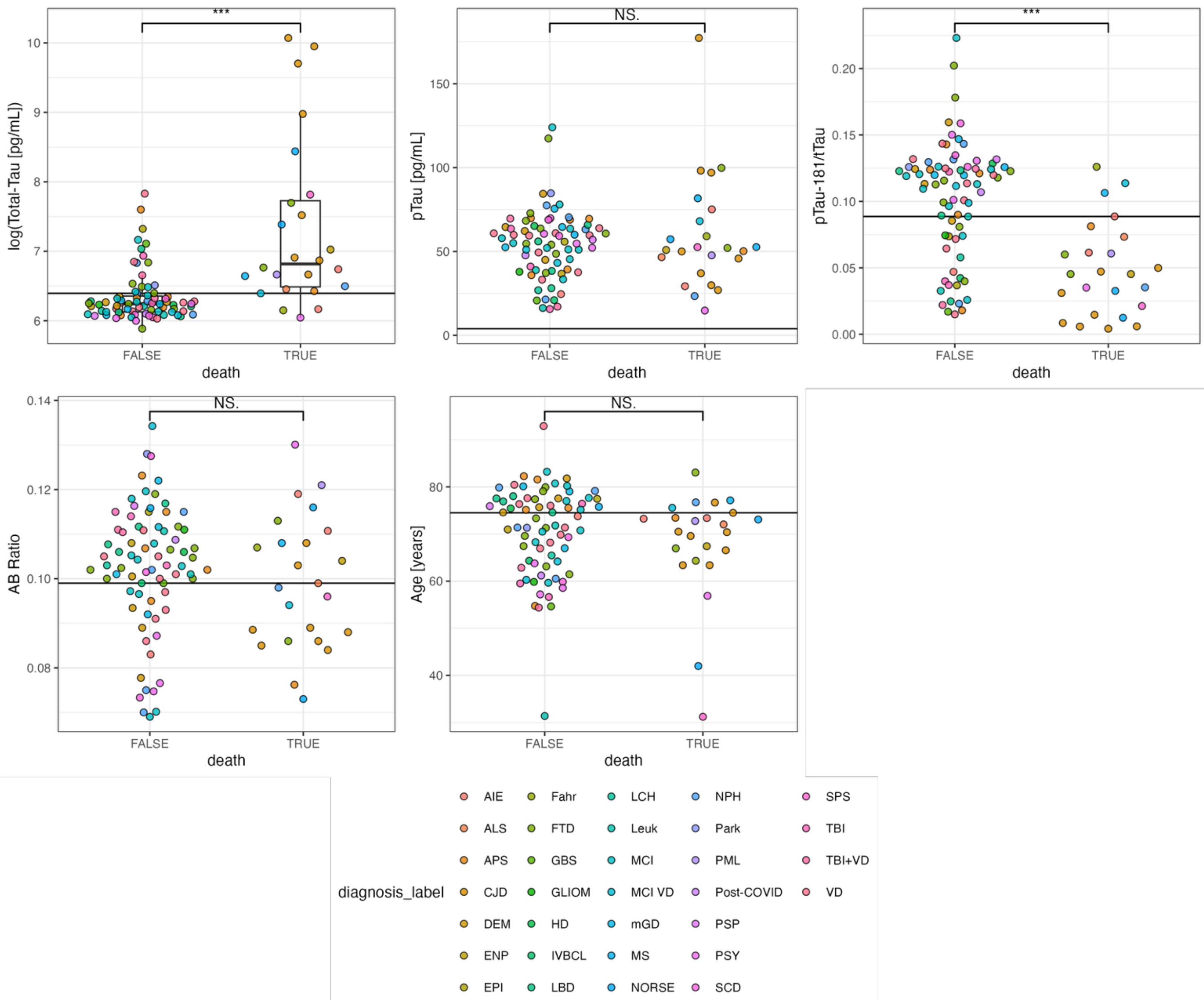

SI 2.5 Biomarker analysis for mortality prediction by diagnosis label

Distribution of tTau (log-transformed), pTau, pTau-181/tTau ratio, Aβ ratio, and age, in survivors vs. deceased patients with SNAP. Wilcoxon rank-sum tests, ***p = 0.001, NS (not significant). Amyotrophic lateral sclerosis (ALS), atypical Parkinsonian syndrome (APS), autoimmune encephalitis (AIE), chronic psychiatric disorder (PSY), dementia/gait disorder — not otherwise specified (DEM), epilepsy (EPI), frontotemporal dementia (FTD), glioma (GLIOM), Guillain-Barré syndrome (GBS), Huntington disease (HD), intravascular B-cell lymphoma (IVBCL), Langerhans cell histiocytosis (LCH), Lewy body disease (LBD), leukoencephalopathy (Leuk), mild cognitive impairment — not otherwise specified (MCI), mild cognitive impairment — vascular (MCI VD), multiple sclerosis (MS), normal pressure hydrocephalus (NPH), not otherwise specified (NOS), new.onset refractory status epilepticus (NORSE), post-COVID (Post-COVID), progressive multifocal leukoencephalopathy (PML), progressive supranuclear palsy (PSP), prion disease/Creutzfeld-Jakob disease (CJD), stiff person syndrome (SPS), subjective cognitive deficits (SCD), traumatic brain injury (TBI), traumatic brain injury with vascular dementia (TBI+VD), encephalopathy syndrome (ENP), vascular dementia (VD).

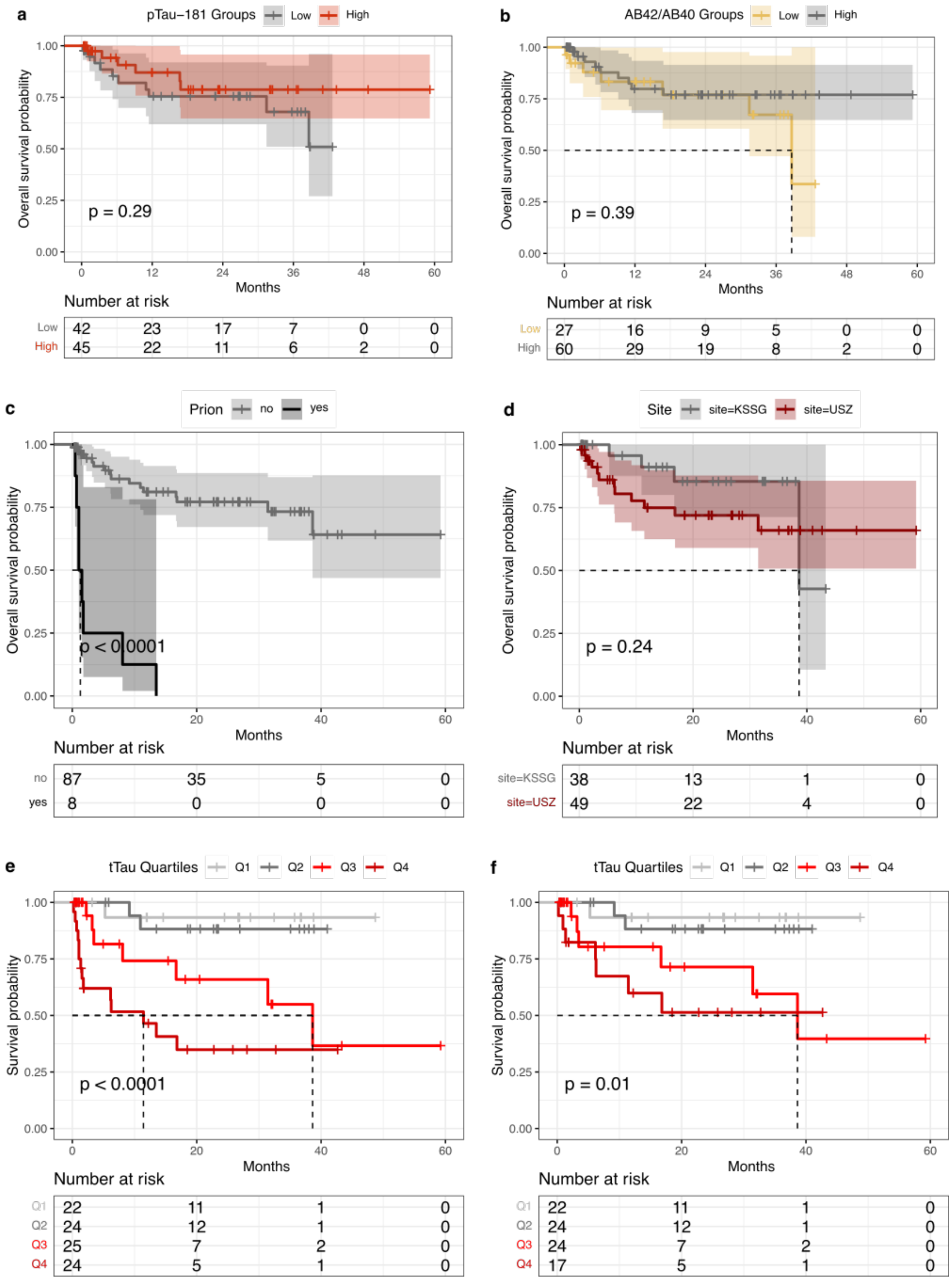

SI 2.6 Survival Analysis for Mortality Prediction in SNAP

a. Kaplan-Meier curves of event-free survival for patients with SNAP divided into pTau and Aβ ratio groups. P-values from log-rank tests. Lower panels indicate numbers at risk. **c-d.** tTau quartiles in SNAP (including prion cases, **c**) and (excluding prion cases, **d**). P-values from log-rank tests. Lower panels indicate numbers at risk.

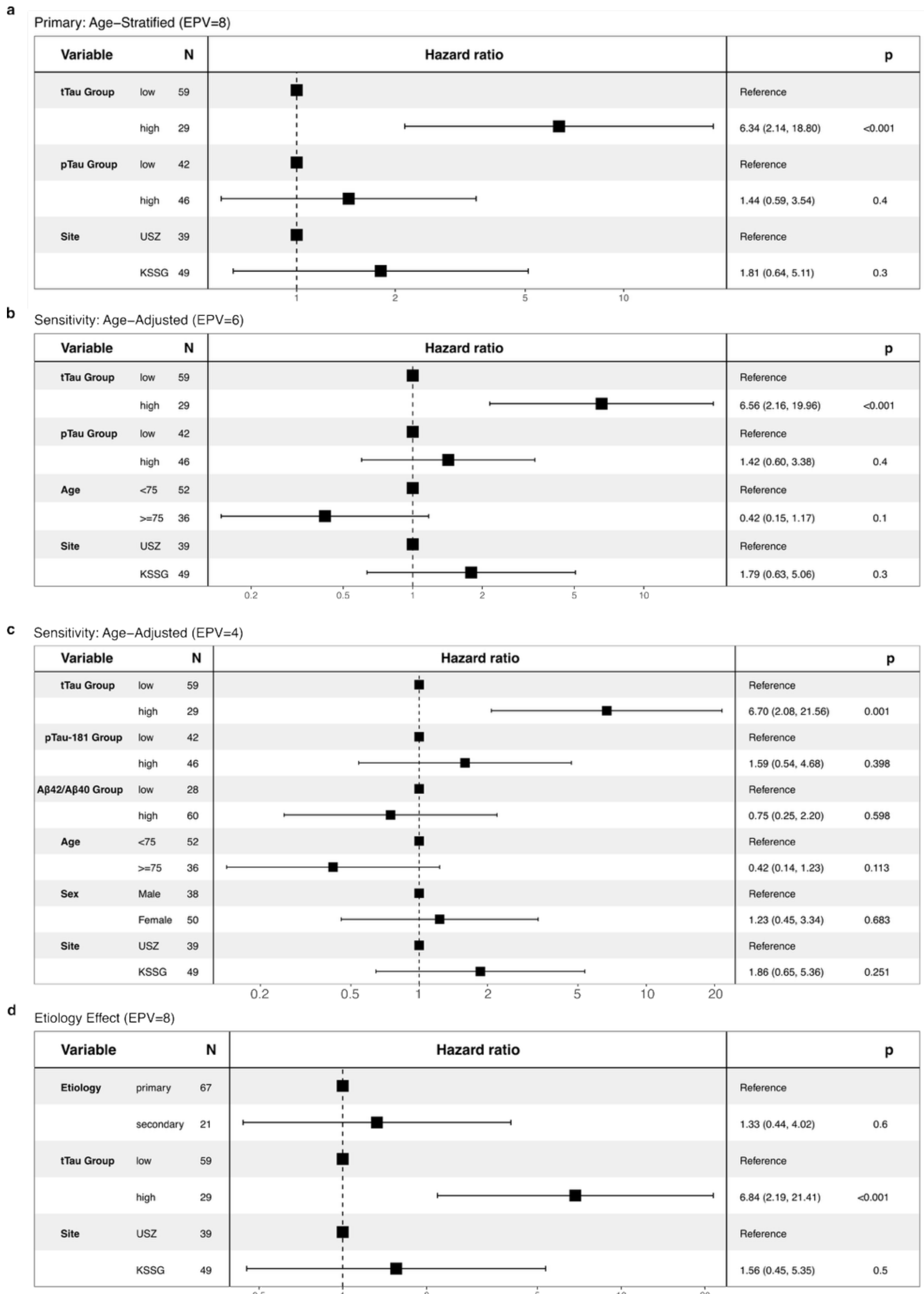

SI 2.7 Cox Hazard Models with Varying Events-Per-Variable (EPV)

a. Results from multivariable Cox proportional hazard model for biomarker groups and site variables (EPV ~ 8). Left column indicates variables, their values and number of available data points in the left column. Middle column indicates forest plot of hazard ratios with mean (box) and confidence interval (whiskers). Right column indicates raw values of hazard ratios and confidence intervals, and p-values. **b.** Results from multivariable Cox proportional hazard model for biomarker groups, age and site variables (EPV ~ 6). Plot structure is same as in a. **c.** Results from multivariable Cox proportional hazard model for biomarker groups, age, sex and site variables (EPV ~ 4). Plot

structure is same as in a. **d.** Results from multivariable Cox proportional hazard model for primary/idiopathic or secondary etiology, tTau group and site variables (EPV ~ 8). Plot structure is same as in a.
